# A Narrative Review of Methodological Considerations in Magnetic Resonance Imaging of Offspring Brain Development and the Influence of Parenting

**DOI:** 10.3389/fnhum.2021.694845

**Published:** 2021-08-19

**Authors:** Shiv Bhanot, Signe Bray, Alexander McGirr, Kate Lee, Daniel C. Kopala-Sibley

**Affiliations:** ^1^Department of Psychiatry, University of Calgary, Calgary, AB, Canada; ^2^Department of Radiology, Cumming School of Medicine, University of Calgary, Calgary, AB, Canada; ^3^Mathison Centre for Mental Health Research and Education, University of Calgary, Calgary, AB, Canada; ^4^Alberta Children’s Hospital Research Institute, Calgary, AB, Canada; ^5^Hotchkiss Brain Institute, Cumming School of Medicine, University of Calgary, Calgary, AB, Canada; ^6^Department of Psychology, York University, Toronto, ON, Canada

**Keywords:** brain development, parenting, MRI, fMRI, child development, adolescence

## Abstract

Parenting has been robustly associated with offspring psychosocial development, and these effects are likely reflected in brain development. This hypothesis is being tested with increasingly rigorous methods and the use of magnetic resonance imaging, a powerful tool for characterizing human brain structure and function. The objective of this narrative review was to examine methodological issues in this field that impact the conclusions that can be drawn and to identify future directions in this field. Studies included were those that examined associations between parenting and offspring brain structure or function. Results show four thematic features in this literature that impact the hypotheses that can be tested, and the conclusions drawn. The first theme is a limited body of studies including repeated sampling of offspring brain structure and function, and therefore an over-reliance on cross-sectional or retrospective associations. The second involves a focus on extremes in early life caregiving, limiting generalizability. The third involves the nature of parenting assessment, predominantly parent- or child-report instead of observational measures which may be more ecologically valid measures of parenting. A closely related fourth consideration is the examination of detrimental versus positive parenting behaviors. While studies with one or more of these thematic limitations provide valuable information, future study design should consider addressing these limitations to determine how parenting shapes offspring brain development.

## Introduction

The brain develops rapidly during infancy, childhood, and adolescence (Belsky and de Haan, 2011; Grayson and Fair, 2017; Tamnes et al., 2018; Vijayakumar et al., 2018), while substantial physical, emotional, and social maturation occurs through dynamic interactions with the environment (Baumrind, 1991; Bradley and Vandell, 2007; McLeod et al., 2007a,b; Waite et al., 2014; Rose et al., 2017). Parents likely play an important role in this process, depending in part on their behavioral interactions with their child. Indeed, adverse parenting influences children’s psychological development in general (Bradley and Vandell, 2007), and their risk for psychopathology in particular (e.g., Collins et al., 2000; McLeod et al., 2007a,b). Interventions that modify parenting also show effects on children’s mental health (Yap et al., 2016).

The protracted development of the brain likely provides maturational windows, or sensitive periods, such that specific parental behaviors may be particularly influential on brain development at certain ages (see Hensch, 2004 for a review). For excellent reviews pertaining to specific parenting practices and the development of specific neural circuits, such as emotion and reward processing circuitry, we refer the reader to recent work dedicated to this question (e.g., Kujawa et al., 2020; Tan et al., 2020). While it is well established that parenting influences offspring psychosocial development, the study of how this is reflected in the brain is still a relatively new endeavor with important challenges and discoveries to be made. Thoughtful study design will be crucial to our ability to understand how, when, and in what ways parental behavior is important for offspring brain development.

Recent years have seen a proliferation of studies using neuroimaging to infer relationships between parenting and offspring brain development. Here, we focus on the magnetic resonance imaging (MRI) literature examining parenting behavior and offspring brain structure and function. This is a rapidly evolving field, and therefore our focus is on study design considerations that have important repercussions on the specific hypotheses that can be tested, and conclusions drawn, regarding influences of parenting on offspring brain development. This is crucial for future study design; while no study design is perfect, based on the extant literature, special consideration should be given to four thematic design features that are currently pervasive. These are (1) an overreliance on cross-sectional or retrospective methodologies, (2) a focus on extremes of early life childcare and adversity as opposed to more normative experiences, (3) measuring parenting behaviors with self-report measures to the exclusion of observational methods, and (4) a focus on negative rather than positive parenting.

## Methods

In this review, we examine the literature on parenting and offspring brain development, including during infancy, childhood, and adolescence. “Brain development” refers to studies in which neuroimaging was conducted at multiple time points, thereby examining change over time in brain structure or function. Where studies were cross-sectional or longitudinal such that parenting was assessed at one time point and neuroimaging was conducted at a later single time point, we refer to associations between parenting and offspring brain structure or function, as these studies cannot examine change over time in brain structure or function. We focus on MRI, both structural and functional, as it can be used to repeatedly sample brain dynamics and structure with high spatial resolution. The papers reviewed here examined brain gray matter including volume and thickness in a range of brain regions while functional studies employed a range of fMRI tasks (e.g., resting state, emotion processing, reward processing, and to name a few) and analytic approaches (evoked responses and functional connectivity). Only a handful of studies have examined diffusion tensor imaging (DTI) and all DTI-based studies to date to our knowledge have focused on extreme adversity in early childhood as oppose to focusing on parenting *per se* (Eluvathingal et al., 2006; Behen et al., 2009; Choi et al., 2009; Govindan et al., 2010; Huang et al., 2012; Hanson et al., 2013). Sheikh et al. (2014) provide one exception, although they examined interactions between parenting and children’s cortisol reactivity and did not report main effects of parenting on offspring white matter structure. DTI-based studies are therefore not included in this review. The literature using other non-invasive tools and approaches has been described elsewhere (Meyer et al., 2015; Bernier et al., 2016; see Maupin et al., 2015).

In this narrative review, dates searched ranged up to May 2021. Databases searched included PubMed, Psyc Info, Psyc Articles, ISI Web of Science, and Google Scholar. Search terms queried in the title, abstract, or keywords included “parenting” or “parent-child relationships” or “developmental predictors” or “developmental influences” or “childhood adversity” or “early adversity” AND “child brain development” or “adolescent brain development” or “offspring brain development” or “child brain function” or “adolescent brain function” or “child brain structure” or “adolescent brain structure” or “offspring brain function” or “offspring brain structure.” Reference sections of retrieved articles were also examined for any relevant publications that were not found in databases. We also include studies that involve parental deprivation, such as studies of children raised in institutional care, given their implications for the effects of a lack of parental caregiving. In total, 82 studies (Table 1) were found that examined relations between either parenting or childhood adversity/stress in general and offspring brain structure or function.

**TABLE 1:** Methodological summary of studies included in this review.

**Citation**	**Cross-sectional versus longitudinal**	**If longitudinal, repeated measures imaging?**	**Functional or structural**	**Parenting measure (parent-report, child report, observational). And was it concurrent or retrospective?**	**Normative parenting versus extreme adversity?**	**Negative versus positive parenting**	**Sample Size**	**Age of offspring at parent assessment**	**Age of child at MRI**	**Age of child at second MRI**
Barbosa et al., 2018	Cross-sectional		Functional	Parent-report	Normative	Negative and positive	88	8–9 years	8–9 years	N/A
Bernier et al., 2018	Longitudinal	No	Structural	Observational, concurrent	Normative	Positive	33	12–15 months	10–11 years	N/A
Boecker et al., 2014	Longitudinal	No	Functional	Self-report	Adversity	Negative	162	3 months to 15 years	Mean = 24.4 years	N/A
Brody et al., 2019	Longitudinal	No	Functional	Self-report	Normative	Positive	119	11–13 years and 16–17 years	25 years	N/A
Burghy et al., 2012	Longitudinal	No	Functional	Parent-report	Normative	Negative	57	1, 4, and 12 months	18 years	N/A
Butterfield et al., 2021	Longitudinal	No	Functional	Self-report	Normative	Positive	30	Mean age: 11.58 years	13.58 years	N/A
Callaghan et al., 2017	Longitudinal	No	Functional	Self-report and observational, concurrent	Normative	Negative and positive	128	12 years	16 years	N/A
Callaghan et al., 2019	Longitudinal	No	Functional	Parent report	Adversity	Negative	102	5–16 years (Time 1); 8–19 years (Time 2)	5–16 years	N/A
Chaplin et al., 2019	Cross-sectional		Functional	Observation, concurrent	Normative	Negative	66	12–14 years	12–14 years	N/A
Choi et al., 2009	Cross-sectional		Structural	Self-report	Adversity	Negative	32	18–25 years	18–25 years	N/A
Dannlowski et al., 2012	Cross-sectional		Structural	Self-report	Adversity	Negative	148	20–57 years	20–57 years	N/A
Dégeilh et al., 2018	Longitudinal	No	Functional	Observational, concurrent	Normative	Positive	28	13–15 months	10 years	N/A
Dillon et al., 2009	Cross-sectional		Functional	Self-report	Adversity	Negative	29	Mean age: 24.58 years (exp); 37.08 years (control)	Mean age: 24.58 years (exp); 37.08 years (control)	N/A
Fareri et al., 2017	Cross-sectional		Functional	Parent report	Adversity	Negative	88	6–18 years	6–18 years	N/A
Fava et al., 2018	Longitudinal	No	Functional	Self-report (parent)	Adversity	Negative	92	<11 years (retrospective)	9–15 years	N/A
Gee et al., 2013	Cross-sectional		Functional	Self-report (parent)	Normative	Negative	89	6.5–17.6 years	6.5–17.6 years	N/A
Goff et al., 2013	Cross-sectional		Functional	Parent report and child report	Adversity	Negative	69	5–15 years	5–15 years	N/A
Graham et al., 2015	Cross-sectional		Functional	Self-report (parent)	Normative	Negative	23	0–12 months	6–12 months	N/A
Guyer et al., 2015	Longitudinal	No	Functional	Self-report (parent)	Normative	Negative	39	7 years	Mean age: 17.89 years	N/A
Hanson et al., 2012	Cross-sectional		Structural	Self-report (child)	Normative	Negative	61	10–13 years	10–13 years	N/A
Hanson et al., 2015a	Cross-sectional		Functional	Self-report	Adversity	Negative	106	11–15 years (Time 1); 13–18 years (Time 2)	11–15 years	13–18 years
Hanson et al., 2015b	Cross-sectional		Structural	Self-report (child)	Adversity	Negative	128	9–14 years	9–14 years	N/A
Heim et al., 2013	Cross-sectional		Structural	Self-report	Adversity	Negative	51	18–25 years	18–25 years	N/A
Herringa et al., 2013	Longitudinal	No	Functional	Self-report (child)	Adversity	Negative	64	18 years	18 years	N/A
Herringa et al., 2016	Longitudinal	No	Functional	Self-report (parent)	Adversity	Negative	132	18–19 years	18–19 years	N/A
Holmes et al., 2018	Longitudinal	No	Functional	Self-report (child)/child report	Normative	Positive	91	11, 12, and 13 years	25 years	N/A
Kok et al., 2015	Longitudinal	No	Structural	Observational	Normative	Positive	191	1–4 years	8 years	N/A
Kopala-Sibley et al., 2020	Longitudinal	No	Functional	Observational, concurrent	Normative	Negative and positive	79	3 years	10 years	N/A
Jiang et al., 2021	Longitudinal		Functional	Self-report, concurrent	Normative	Negative and positive	89	Mean age: 12.67 years	Mean age: 16.03 years	
Lee et al., 2014	Cross-sectional		Functional	Self-report	Normative	Negative	32	9–17 years	9–17 years	N/A
Lee et al., 2016	Cross-sectional		Structural	Self-report	Adversity	Negative	31	Mean age: 16.12 years	Mean age: 16.12 years	N/A
Lee et al., 2018	Cross-sectional		Structural	Self-report (child)	Adversity	Negative	31	Mean age: 16.12 years	Mean age: 16.12 years	N/A
Luby et al., 2012	Longitudinal	No	Structural	Parent-report and observation, concurrent	Normative	Positive	92	3–5 years; 4–7 years	7–13 years	N/A
Luby et al., 2013	Longitudinal	No	Structural	Observation, concurrent	Normative	Positive	145	Time 1: 3–6 years; Time 2: 6–7 years; Time 3: 8–12 years	Time 1: 10.53 years; Time 2: 12.03 years; Time 3: 13.35 years; Time 4: 16.61 years	N/A
Lupien et al., 2011	Cross-sectional		Structural	Self-report	Normative	Negative	38	13 years	10 years	N/A
McCrory et al., 2011	Cross-sectional		Functional	Self-report	Adversity	Negative	43	12 years	12 years	N/A
McCrory et al., 2013	Cross-sectional		Functional	Self-report	Adversity	Negative	41	12 years	12 years	N/A
McLaughlin et al., 2014	Cross-sectional		Structural	Observation (?) – institutionalized	Adversity	Negative	80	6–30 months	8–10 years	N/A
Mehta et al., 2009	Cross-sectional		Structural	Self-report	Adversity	Negative	14	Mean = 16 years	16 years	N/A
Merz et al., 2019	Cross-sectional		Structural	Self-report (parent)	Normative	Negative	66	5–9 years	5–9 years	N/A
Morgan et al., 2014	Longitudinal	No	Functional	Self-report (parent) and observation, concurrent	Normative	Positive	120	18 and 24 months; 10–11 years	20 years	N/A
Mueller et al., 2010	Cross-sectional		Functional	Observation (?) – institutionalized	Adversity	Negative	33	13 years	13 years	N/A
Ohashi et al., 2017	Cross-sectional		Structural	Self-report	Adversity	Negative	122	18–25 years	18–25 years	N/A
Pagliaccio et al., 2015	Longitudinal	No	Functional	Parent report	Adversity	Negative	120	9–14 years	9–14 years	N/A
Philip et al., 2013a	Cross-sectional		Functional	Self-report (child)	Adversity	Negative	27	Exp: 37 ± 10 years; control: 30 + 9 years	Exp: 37 ± 10 years; Control: 30 ± 9 years	N/A
Philip et al., 2013b	Cross-sectional		Functional	Self-report	Adversity	Negative	21	Mean age: 39.7 years (exp); 37.4 years (control)	Mean age: 39.7 years (exp); 37.4 years (control)	N/A
Pozzi et al., 2019	Cross sectional		Functional	Observational, concurrent and self-report (parent)	Normative	Negative	80	8–9 years	9–10 years	N/A
Pozzi et al., 2020	Longitudinal	No	Functional	Observational, concurrent and parent/child- report	Normative	Positive and negative	86	Time 1: 8–9 years; Time 2: 9–10 years	9–10 years	N/A
Pozzi et al., 2021	Longitudinal	Yes	Functional	Observational	Normative	Positive and negative	95	Mean age: 8.4 years	Mean age: 8.4 years	Mean age: 9.9 years
Rao et al., 2010	Longitudinal	No	Structural	Self-report	Normative	Positive	49	Time 1: 4 years; Time 2: 8 years	13–16 years	N/A
Richmond et al., 2019	Cross sectional		Structural	Observational, concurrent	Normative	Positive and negative	145	8 years	8 years	N/A
Romund et al., 2016	Cross-sectional		Functional	Self-report (child)	Normative	Positive	83	13–16 years	13–16 years	N/A
Roth et al., 2018	Cross-sectional		Structural	Self-report	Adversity	Negative	138	9–15 years	9–15 years	N/A
Schneider et al., 2012	Cross-sectional		Structural and functional	Self-report	Normative	Positive	63	Mean age: 14.24 years	Mean age: 14.24 years	N/A
Sheridan et al., 2012	Cross-sectional		Structural	Observation (?) – institutionalized	Adversity	Negative	74	8–11 years	8–11 years	N/A
Soe et al., 2016	Longitudinal	No	Functional	Self-report (parent)/parent report	Normative	Negative	258	6, 18, and 24 months	6 and 18 months	N/A
Stein et al., 1997	Cross-sectional		Structural	Self-report	Adversity	Negative	21	32.0 years	32 years	N/A
Suffren et al., 2021	Longitudinal	No	Structural	Parent-report	Adversity	Negative	94	2.5–9 years	12–16 years	N/A
Taylor et al., 2006	Cross-sectional		Functional	Self-report	Normative	Negative	30	18–36 years	18–36 years	N/A
Teicher et al., 2004	Cross-sectional		Structural	Self-report	Adversity	Negative	166	Mean age: 12.9 years (exp); 11.9 years (control)	Mean age: 12.9 years (exp); 11.9 years (control)	N/A
Thijssen et al., 2017	Cross-sectional		Functional	Observation, concurrent	Normative	Negative	124	6–10 years	6–10 years	N/A
Thomason et al., 2015	Cross-sectional		Functional	Self-report (child)	Adversity	Negative	42	9–15 years	9–15 years	N/A
Tomoda et al., 2009	Cross-sectional		Structural	Self-report	Normative	Negative	45	18–25 years	18–25 years	N/A
Tomoda et al., 2011	Cross-sectional		Structural	Self-report	Normative	Negative	21	18–25 years	18–25 years	N/A
Tottenham et al., 2010	Cross-sectional		Structural	Self-report	Adversity	Negative	78	Mean age: 60–134 months	Mean age: 62–142 months	N/A
Tottenham et al., 2011	Cross-sectional		Functional	Observation, concurrent	Adversity	Negative	44	Mean age: 9 years (exp); 10 years (control)	Mean age: 9 years (exp); 10 years (control)	N/A
Tyborowska et al., 2018	Longitudinal	No	Structural	Observational, concurrent and parent report	Normative	Negative	37	17 years	14 years (Time 1) and 17 years (Time 2) years	N/A
Van der Werff et al., 2013	Cross-sectional		Functional	Self-report	Adversity	Negative	88	39 years	39.0	N/A
Vidal-Ribas et al., 2019	Longitudinal	No	Functional	Parent-report	Normative	Negative-early life stress (e.g., sickness, exposure to violence and family accomplishments)	38	7 years	10 years	N/A
Walsh et al., 2014	Cross-sectional		Structural	Self report	Adversity	Negative	58	17–20 years	17–20 years	N/A
Wang et al., 2019	Longitudinal	Yes	Functional	Observational, concurrent	Normative	Positive	137	6 months	4 and 6 years	N/A
Whittle et al., 2008	Cross-sectional		Structural	Observational, concurrent	Normative	Negative	137	11.4–13.7 years	11.4–13.7 years	N/A
Whittle et al., 2009	Cross-sectional		Structural	Observational, concurrent	Normative	Negative	113	11.4–13.6 years	11.4–13.6 years	N/A
Whittle et al., 2011	Cross-Sectional		Structural	Observational, concurrent	Adversity	Negative	?	Mean = 12.2 years	Mean = 12.2 years	N/A
Whittle et al., 2013b	Longitudinal	Yes	Structural	Observational, concurrent	Normative	Positive	188	12 years	12 years	16 years
Whittle et al., 2013a	Longitudinal	Yes	Structural	Self-report	Adversity	Negative	117	Mean age: 12.6 years (Time 1); 16.4 years (Time 2)	Mean age: 12.6 years	16.4 years
Whittle et al., 2016	Longitudinal	Yes	Structural	Observational, concurrent	Normative	Negative	166	12 years	12 years	16 years (Time 2); 19 years (Time 3)
Whittle et al., 2017	Longitudinal	Yes	Structural	Observational, concurrent	Normative	Positive	177	13 years	13 years	17 years (Time 2); 19 years (Time 3)
Williamson et al., 2009	Cross sectional		Functional	Self-report (child)	Adversity	Negative	32	20–53 years (exp); 21–59 years (control)	20–53 years (exp); 21–59 years (control)	N/A
Wolf and Herringa, 2016	Cross sectional		Functional	PTSD diagnosis (?)	Adversity	Negative	53	8–18 years	8–18 years	N/A
Yap et al., 2008	Cross-Sectional		Structural	Observational, concurrent	Normative	Negative	106	11.4–13.6 years	11.4–13.6 years	N/A
Zhu et al., 2019	Cross-sectional		Functional	Self-report (child)	Adversity	Negative	202	20–25 years	20–25 years	N/A

A common consideration across many of these studies is that the primary purpose of the research programs from which the data is drawn was not to examine parenting and offspring brain development, *per se*. Instead, parenting was an important secondary endpoint in studies of risk for psychopathology in children and youth.

## Results

### Cross-Sectional Versus Longitudinal Studies of Parenting and Offspring Brain Development

Given substantial inter-individual differences in brain structure and function, longitudinal designs that control for baseline characteristics are the gold standard for studying developmental processes rather than cross-sectional associations (Crone and Elzinga, 2015). The methods of the studies reviewed here are summarized in Table 1. The majority of the literature on the associations of parenting with child brain structure or function is cross-sectional. Few studies have conducted repeated measures of brain imaging. Indeed, of the studies surveyed (Table 1), 47 (57.32%) were cross sectional. Of the papers containing longitudinal data (10 structural MRI and 23 fMRI), only 12 (14.63% of all studies) assessed parenting and brain structure or function and then conducted MRI scans at a later time point (Whittle et al., 2013a,b, 2016, 2017; Kok et al., 2015; Pagliaccio et al., 2015; Tyborowska et al., 2018; Wang et al., 2019; Butterfield et al., 2021; Jiang et al., 2021; Pozzi et al., 2021; Suffren et al., 2021), although only five examined brain structural (Whittle et al., 2013a,b, 2016, 2017) or functional development over time (Pozzi et al., 2021).

Cross-sectional studies or studies where parenting is assessed at one time point and brain structure or function assessed at a single later time point provide valuable associations, but they have important limitations. This is particularly so in the study of development, where the research question is related to change in brain structure or function over time. Many associations between parenting and offspring brain structure or function will likely be replicated when subjected to longitudinal repeated measures study designs, however important confounds identified in the developmental psychology literature may also apply to the parenting and offspring brain development literature. For instance, it is well-established that the parent-child relationship is bi-directional and children have a substantial influence on the parenting they receive (Belsky, 1984; Burke et al., 2008; Pardini et al., 2008; Combs-Ronto et al., 2009; Hawes et al., 2011; Kopala-Sibley et al., 2017). Moreover, given that parenting styles and behaviors tend to be relatively stable over time (e.g., Dallaire and Weinraub, 2005; Kopala-Sibley et al., 2017), it will be important to carefully consider when to measure parenting and offspring brain structure and function to separate acute from persistent effects. By not assessing children’s brain structure or function at baseline concurrently with the parenting assessment, effects of parenting at baseline on later brain structure or function could be due to a baseline association that has not been accounted for.

A closely related issue is that longitudinal studies with multiple imaging time points are necessary to assess brain development trajectories. Given the marked changes in brain structure and function across development, the interpretation of an association between parenting and brain structure in childhood might be very different than in adolescence. For example, adverse parenting may be associated with reduced cortical thickness in childhood but increased cortical thickness in adolescence. Longitudinal analyses of multiple timepoints of imaging data would be required to test the possibility that while adverse parenting is associated with reduced cortical thickness in childhood, it is also associated with a slower pattern of thinning during adolescence, in contrast to the normal developmental pattern of cortical thickness (Vijayakumar et al., 2016).

### Retrospective Child- or Parent-Report Versus Observational Measures of Parenting

An exclusive focus on child- or parent-reports is an issue in a wide range of studies examining associations between parenting and offspring brain structure or function as assessed via MRI or fMRI. Indeed, of the studies examined (Table 1), the large majority employed retrospective self-report measures, while 27 (32.92%, including studies of institutionalized children) utilized observational measures such as a videotaped interaction tasks performed in a laboratory setting. Of the studies employing observational methods (Table 1), 15 (18.29% of all studies) examined brain structure and 12 (14.63% of all studies) examined brain function. Of these 12 that employed an observational approach to measuring parenting and examined brain function, five (6.25% of all studies) involved longitudinal assessment of parenting at one time point and brain function at a later time point (Morgan et al., 2014; Dégeilh et al., 2018; Wang et al., 2019; Kopala-Sibley et al., 2020; Pozzi et al., 2021).

The major design considerations with self-reported parenting, particularly retrospective self-report, is the confounding effect of factors such as the current mood or mental health of the respondent, the interval between the occurrence of an event and the reporting of that event, and interpersonal factors that have since occurred between the parent and child (see Hardt and Rutter, 2004). It should be noted that while concurrent as opposed to retrospective self-reports are likely less influenced by memory issues, they may still be influenced by current mood, mental health, or recent interpersonal factors between the parent and child. This is not limited to parental self-reports, as child and parent reports of parenting do not correspond well with each other (Sessa et al., 2001; Zaslow et al., 2006). A large body of literature has called into question the validity of retrospective reports of experiences, as these corroborate poorly with contemporaneous assessment and documentation (Yarrow et al., 1970; Brewin et al., 1993; Henry et al., 1994; Hardt and Rutter, 2004).

A recent meta-analysis (Baldwin et al., 2019) reviewed 16 studies comprising 25,471 participants. These were studies in which children and youth had been followed over time and had concurrent, documented histories of experiencing maltreatment, as determined by child protective services, police records, or parent, child, or teacher reports. Across these studies, youth also completed retrospective self-reports of recalled maltreatment experiences. Baldwin et al. (2019) then examined the concordance between prospective and retrospective accounts of maltreatment. They found very poor agreement (kappa = 0.18) and concluded that prospective and retrospective accounts of maltreatment identify different groups of individuals. More troubling, 52% of youth with contemporaneously documented histories of maltreatment did not retrospectively report it, while 56% of youth who retrospectively reported maltreatment did not have concordant prospective observations. Associations between current brain structure and function and self-reported accounts of developmental experiences, particularly retrospectively, should be interpreted with caution.

A possible solution to this issue is the use of laboratory and/or in-home observations of parent child interactions to quantify parenting behavior. Indeed, laboratory-based observations of parent-child interactions tend to correspond well with in-home observations of parent-child interactions (Zaslow et al., 2006). There is also evidence that home and laboratory observational measures are better predictors of child psychosocial outcomes, including social skills and academic achievement, than parent or child reports (Zaslow et al., 2006). While laboratory or home-based assessments provide unique data on dyadic interactions, they are also subject to limitations including ecological validity, transient influences such as mood states, and restrictions in the range of affect and behavior elicited in the parent and child. It is therefore likely that parent-reports and laboratory-based observations provide complementary information on parent-child relationships. Future research will likely benefit from examining how different measures of parenting converge or differ in their associations with offspring brain structure or function.

### Extreme Forms of Adversity Versus Normative Experiences

Numerous studies focused on extreme forms of developmental adversity, such as abuse, neglect, or institutional rearing. This is the case in 39 studies (47.56%, Table 1). What is unclear is whether parenting behavior lies on a continuum, and whether a parametric relationship exists along this continuum in order to inform the influences of normative parenting on brain structure and function. Most children will not experience maltreatment (Wildeman et al., 2014), however all children will receive both positive and negative parenting with varying frequency and intensity. Our knowledge of how the parenting experiences of most children affect brain structure or function is somewhat limited. To date, 43 studies (52.43%, Table 1) studies have examined the effects of normative parenting in infants, children, or adolescents (e.g., Whittle et al., 2008, 2009; Wildeman et al., 2014; Whittle et al., 2016, 2017; Yap et al., 2008; Schneider et al., 2012; Lee et al., 2014; Guyer et al., 2015; Kok et al., 2015; Romund et al., 2016; Thijssen et al., 2017; Bernier et al., 2018; Dégeilh et al., 2018; Brody et al., 2019; Wang et al., 2019; Kopala-Sibley et al., 2020; Butterfield et al., 2021; Jiang et al., 2021). Twenty-three of these 28 (75%) were longitudinal in that they either measured parenting at one time point and brain structure or function at a later time point, or they measured parenting at baseline and then included repeated measures of neuroimaging.

This is important, because non-pathological behaviors have been associated with offspring differences in brain structure and function, including variability in parenting styles (Guyer et al., 2015; Dégeilh et al., 2018), maternal hostility (Kopala-Sibley et al., 2020), aggression (Whittle et al., 2013b, 2016), and behavioral regulation (Kopala-Sibley et al., 2020). Further research examining non-pathological parental behavior will be important to determining dose-response relationships between parental behavior and offspring brain development, and how extremes in early life caregiving behaviors can inform our knowledge of potential effects of normative parenting. It will also be important in future research to examine whether adversity versus normative parenting are uniquely associated with offspring brain development.

### A Focus on Negative Versus Positive Parenting

The majority of research to date has focused on negative parenting behaviors (Table 1). Twenty-two (26.83%) studies have examined positive parenting, of which nine (10.97% of all studies) were structural and 14 (17.07% of all studies) were functional (some included both structure and function and/or both positive and negative parenting). Of the nine structural studies, eight were longitudinal (9.76% of all studies), but only four examined brain structure at multiple time points (Whittle et al., 2013a,b, 2016, 2017; 4.82% of all studies). Of the 14 functional studies, 11 were longitudinal (12.99% of all studies), with one study to date examining change over time in brain function (Pozzi et al., 2021).

This is an important knowledge gap in understanding the impact of parental behaviors on offspring brain structure and function. Positive behaviors including warm, sensitive, and supportive parenting behaviors have been associated with a range of improved psychosocial outcomes in offspring including improved academic performance, more adaptive temperament, and lowered risk for psychopathology (Beckwith et al., 1992; Eshel et al., 2006; Landry et al., 2008). Moreover, positive and negative parenting are not opposite sides of the same coin and do not necessarily lie on a continuum. For example, a lack of negative parenting (e.g., harshness, criticism, and control) does not necessarily imply high levels of positive parenting. Multiple levels of positive and negative parenting may exist together in various combinations and permutations and may be at least somewhat orthogonal (Whittle et al., 2014). The unique contributions of positive and negative parenting behaviors, and their synergies, will be an important design consideration for future studies examining the effects of parenting on offspring brain structure and function.

## Discussion

We reviewed thematic design features that have important scientific ramifications for our understanding of how parenting influences offspring brain structure and function. We also include studies examining developmental adversity in general as these studies, while not focusing directly on parenting, typically include adverse experiences with parents (e.g., maltreatment or neglect) in their measures of adversity. The purpose of this review is to highlight areas within this rapidly evolving field that would benefit from next-generation approaches and methodologies. When possible, future studies should consider longitudinal, multi-wave acquisitions of both parenting and offspring neuroimaging, the inclusion of observational assessments and normative parenting behaviors, as well as consideration of positive as well as negative parenting behaviors.

Though many of the studies we review were not originally designed to examine parenting and offspring brain structure or function *per se*, they have provided important insights into potential influences of parenting on offspring brain development. In particular, of the various brain regions examined in the studies included in this review, the majority have focused on subcortical, including limbic, striatal, and hippocampal regions, as well as prefrontal cortical regions (e.g., Whittle et al., 2013a; Morgan et al., 2014; Pagliaccio et al., 2015; Wang et al., 2019; Kopala-Sibley et al., 2020; Pozzi et al., 2020; Butterfield et al., 2021). This may be in part because at least some of these studies stemmed from broader cohort studies whose primary focus was understanding child and adolescent risk factors for adverse behavioral outcomes, in particular psychopathology such as depression and anxiety, as well as effects of parenting on child emotional and behavioral regulation (e.g., Whittle et al., 2013a, 2016; Pagliaccio et al., 2015). Thus, these regions may have been the focus of numerous studies given links between their structure or function and child mental health and behavior.

However, studies have also found associations between parenting in infancy, childhood, or adolescence and global cortical thickness (Frye et al., 2010), total gray matter volume (Kok et al., 2015), functioning in the occipital lobe in adolescents (Pozzi et al., 2020), functional connectivity in corticolimbic regions (Jiang et al., 2021), and large-scale functional brain networks such as functional connectivity of the default mode and salience networks in late childhood or adolescence (Graham et al., 2015; Dégeilh et al., 2018; Pozzi et al., 2021). Research confirms associations between self-reported and retrospectively recalled adverse developmental experiences and suboptimal parenting and offspring brain structure and function, in particular in limbic, striatal, and prefrontal region structure, function, and functional connectivity during a range of fMRI tasks. A smaller, but rapidly growing, body of evidence suggests that parenting at one time point, such as early childhood or adolescence, is associated with brain function in emotion and reward processing regions at a later time point, even as much as eight (Kopala-Sibley et al., 2020) to 20 years (Morgan et al., 2014) later. Other studies have confirmed links between parenting in infancy or early childhood and function in large scale brain networks later in childhood (Dégeilh et al., 2018) and regions linked to memory, stress, and affect processes (Wang et al., 2019). These studies suggest a potentially long-lasting impact of parenting on offspring brain function. Only three studies of which we are aware (Whittle et al., 2014, 2016; Pozzi et al., 2021) showed that parenting, observationally assessed within a normative range during adolescence, predict brain development. Consistent with prior research suggesting a longitudinal link between parenting and limbic and prefrontal brain structure or function, Whittle et al. (2013b), Whittle et al. (2016) confirm that parenting predicts change over time in amygdala and prefrontal brain structure. Recently, Pozzi et al. (2021) show that parenting predicts change over time in resting state functional connectivity in older children.

Results to date may also have implications for our understanding of the importance of developmental timing in terms of the effects of parenting on offspring brain development. The handful of studies that have examined parenting and brain structure or function in infants and children (Graham et al., 2015; Soe et al., 2016; Bernier et al., 2018; Dégeilh et al., 2018; Wang et al., 2019; Kopala-Sibley et al., 2020) confirm postulations that this age-range is a period during which the developing brain may be particularly vulnerable to developmental insults (Teicher et al., 2018; Luby et al., 2019). However, results from the current review confirm an association between parenting and late childhood and adolescent brain structure and function, as well (Teicher et al., 2018; Luby et al., 2019). This highlights the need for dedicated research efforts throughout development to understand the impacts of parenting on offspring brain structure and function at different developmental stages.

### Challenges in Dissecting Parenting Effects on Offspring Brain Development

There are unique challenges to this field of research. First, it requires following a relatively large cohort in which both parenting and child brain structure or function are assessed at multiple time points over the course of development. This will be necessary to examine associations between parenting and trajectories of offspring brain development. Assessing parenting and offspring brain structure of function at multiple time points will also be necessary to examine potential bi-directional associations between parenting and offspring brain development. This is inherently difficult given challenges around recruitment and retention, especially when both parent and child need to participate, although some evidence suggests that longitudinal designs are more efficient for the study of change in brain structure, suggesting that smaller sample sizes may be adequate (Steen et al., 2007).

Second, MRI scans are costly, and pediatric MRI poses substantial challenges given factors such as that children may move more in the scanner relative to adults, thereby making some data unusable, while other children may be claustrophobic, for example. Dental hardware provides another challenge in MRI in youth, as this can cause significant artifacts. The study of brain development requires at least two scans over time per participant to model between-subject rank-order change (i.e., a change in a participant’s standing relative to other participants), and at least three scans to model within-subject linear change (i.e., individual participant’s linear within-subject change). Indeed, four scans or would be required to model non-linear (e.g., quadratic; see King et al., 2018 for a review of longitudinal modeling methods of developmental neuroscience research). It is well-established that many brain regions do not develop linearly (e.g., Vijayakumar et al., 2016), and parenting may influence brain development in a non-linear manner. It will be important to examine this possibility in future research, although it would likely be quite costly.

Third, fMRI tasks designed to elicit certain cognitive processes in the brain (e.g., emotion processing), may not be appropriate or repeatable at different developmental stages, rendering examination of long-term change in specific aspects of brain function difficult as these may require different tasks at different ages. This is a common challenge in developmental research as assessing within-subject change requires identical measurement methods at each time point, although measures appropriate for one age are often not appropriate for another.

Fourth, while observational assessments of parenting are arguably the gold-standard method of assessing parenting, these are time consuming to conduct and to code, with coders requiring extensive training. There are also multiple factors other than parenting such as socioeconomic status, community violence, school quality, and sibling and peer relationships, to name a few, that may affect brain development or interact with parenting to affect brain development.

Finally, some consideration should be given to which parent participates in research. The vast majority of this literature has examined effects of maternal rather than paternal parenting, as is common in developmental psychology research (Parent et al., 2017). Until proven otherwise, it may be appropriate to recognize that the existing literature is informative of “mothering,” as opposed to “parenting.” Nevertheless, paternal behaviors are robustly linked to children’s psychopathological outcomes (see Möller et al., 2016 for a meta-analysis), and it will therefore be important to understand convergence and divergence between maternal and paternal influences on offspring brain structure and function.

## Other Considerations for Future Study Design

Although noted by Belsky and de Haan (2011), there have yet to be any studies examining reciprocal or bi-directional relationships between parenting and offspring brain development. Indeed, it is well-established that children influence the environment around them (Belsky, 1984; Belsky and Jaffee, 2006). There is also substantial evidence that while parenting influences children’s behavior and mental health, children’s behavior and psychopathological symptoms influence parenting (Kopala-Sibley et al., 2017). These effects and behaviors will be mediated via children’s brain development; however, no research has tested this possibility.

Relatedly, there is substantial evidence that parenting behaviors change over time. For example, maternal harsh parenting toward their infant or young child tends to increase from birth through the age of 3 (Kim et al., 2010), while overreactive parenting increases and parenting self-efficacy decreases over the first few years of a child’s life (Lipscomb et al., 2011). Similarly, parental support tends to decrease over the course of adolescence (Wang et al., 2011), although there will be substantial variability in these trajectories across parents. However, it is unknown how within-subject trajectories of parenting, as opposed to between-subject differences, relate to offspring brain development.

Another important consideration is whether to diverge from a naturalistic design toward an experimental one. A substantial body of evidence has found that interventions meant to ameliorate parenting are effective in improving youth outcomes such as mood and anxiety disorders (see Yap et al., 2016 for a meta-analysis) as well as externalizing symptoms (Kazdin, 2005; Ogden and Hagen, 2008). Using these interventions as a tool to examine effects on offspring brain structure and function may be a more cost-effective means of determining associations with causal inference. Yet, solid naturalistic data will be required to determine the optimal periods of development, tasks, and inter-sample interval required for such experimental designs.

## Conclusion

We reviewed the current literature examining parenting and offspring brain structure or function as well as studies examining offspring brain development over time with a focus on MRI. We identify four thematic design features that have important effects on the hypotheses that can be tested and constrain the possible interpretations of how parenting impacts offspring brain development. As a developmental field, we are primarily interested in change in brain structure and function over time, yet there remains a dearth of repeated measures longitudinal data. This rapidly evolving field is incorporating increasingly rigorous methodologies, and future studies should also consider multiple means of assessing parenting behaviors, including positive parenting behaviors, and testing whether normative parenting and pathological early life adversity lie on a continuum. Studies incorporating these considerations will constitute next generation designs in the field of parenting and offspring brain development.

## Author Contributions

SBh conducted the literature review and contributed to the writing of this manuscript. SBr and AM provided invaluable consultation on interpretation of results and manuscript preparation. KL facilitated building Table 1 and collating results and provided edits to the manuscript. DK-S was the principal investigator of this study and contributed to writing the manuscript. All authors contributed to the article and approved the submitted version.

## Conflict of Interest

The authors declare that the research was conducted in the absence of any commercial or financial relationships that could be construed as a potential conflict of interest.

## Publisher’s Note

All claims expressed in this article are solely those of the authors and do not necessarily represent those of their affiliated organizations, or those of the publisher, the editors and the reviewers. Any product that may be evaluated in this article, or claim that may be made by its manufacturer, is not guaranteed or endorsed by the publisher.
